# Coronavirus disease 2019 (COVID-19) and the renin-angiotensin system: A closer look at angiotensin-converting enzyme 2 (ACE2)

**DOI:** 10.1177/0004563220928361

**Published:** 2020-06-02

**Authors:** Annalise E Zemlin, Owen J Wiese

**Affiliations:** Department of Pathology, Chemical Pathology Division, National Health Laboratory Service (NHLS) and University of Stellenbosch, Tygerberg Hospital, Cape Town, South Africa

**Keywords:** COVID-19, SARS-CoV-2, angiotensin-converting enzyme 2, renin-angiotensin system

## Abstract

Since the first cases of atypical pneumonia linked to the Huanan Seafood Wholesale Market in Wuhan, China, were described in late December 2019, the global landscape has changed radically. In March 2020, the World Health Organization declared COVID-19 a global pandemic, and at the time of writing this review, just over three million individuals have been infected with more than 200,000 deaths globally. Numerous countries are in ‘lockdown’, social distancing is the new norm, even the most advanced healthcare systems are under pressure, and a global economic recession seems inevitable. A novel coronavirus (SARS-CoV-2) was identified as the aetiological agent. From experience with previous coronavirus epidemics, namely the severe acute respiratory syndrome (SARS) and Middle East Respiratory Syndrome (MERS) in 2004 and 2012 respectively, it was postulated that the angiotensin-converting enzyme-2 (ACE2) receptor is a possible port of cell entry. ACE2 is part of the renin-angiotensin system and is also associated with lung and cardiovascular disorders and inflammation. Recent studies have confirmed that ACE2 is the port of entry for SARS-CoV-2. Male sex, advanced age and a number of associated comorbidities have been identified as risk factors for infection with COVID-19. Many high-risk COVID-19 patients with comorbidities are on ACE inhibitors and angiotensin receptor blockers, and this has sparked debate about whether to continue these treatment regimes. Attention has also shifted to ACE2 being a target for future therapies or vaccines against COVID-19. In this review, we discuss COVID-19 and its complex relationship with ACE2.

## Introduction

In these unprecedented times, the Coronavirus disease 2019 (COVID-19) pandemic has had a severe impact globally. Most countries have implemented strict ‘lockdown’, and social distancing and isolation of the elderly and vulnerable are advised. At the time of writing this review, just over three million individuals have been affected globally with more than 200,000 deaths.^1^ The global infection curve has not flattened, with new infections continuing to rise. Although the pandemic originated in China, the epicentre shifted to Europe, and presently the most affected country is the United States of America (USA).

## COVID-19

### Timeline

**Figure 1.**
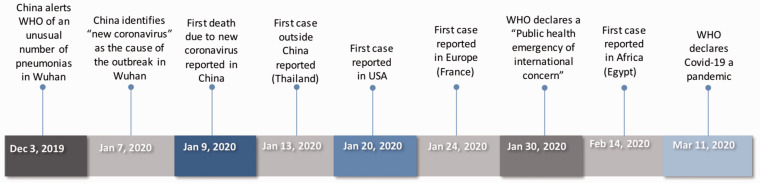
A timeline illustrating the most notable events of the COVID-19 outbreak.Source: World Health Organization (WHO).

The first cases of atypical pneumonia linked to the Huanan Seafood Wholesale Market in Wuhan, China were described in late December 2019. China announced an outbreak on 31 December 2019 and closed the market once it became apparent that this was the place of origin. In early January 2020, investigations by Chinese authorities continued, contacts were traced and managed and the World Health Organization (WHO) was notified. On 7 January 2020, a novel coronavirus was announced as the cause of the outbreak,^2^ and on 10 January 2020, China shared the viral gene sequence to allow the development of reverse transcriptase-polymerase chain reaction (RT-PCR) diagnostic kits.^3^ The first case outside China was confirmed in Thailand, and strict screening measures, travel restrictions and the lockdown of Wuhan were implemented.^4^ The first case in the USA was announced on 20 January 2020^5^ and the first cases in Europe were reported in France on 24 January 2020 in individuals with a travel history to the Hubei Province, China. Subsequent human-to-human transmission followed, and cases with no travel history were reported.^6^ Africa reported its first case on 14 February 2020 in Egypt. Since then, the number of cases has risen dramatically (Figure 1).

### Epidemiology and pathogenesis

This is the third coronavirus outbreak in as many decades.^7^ The previous two were severe acute respiratory syndrome (SARS) in 2003, and Middle East respiratory syndrome (MERS) in 2012 which had mortality rates of 10% and 37%, respectively.^8^ The mortality rate of SARS-CoV-2, – although lower compared to SARS and MERS, seems to have a more devastating effect globally.

The SARS-CoV-2 has a single-stranded RNA genome enveloped in a lipid membrane embedded with glycoprotein spikes giving the impression of a crown – hence the name coronavirus (CoV). RNA viruses use an error-prone RNA polymerase for replication and therefore have high mutation rates and can easily mutate during epidemics adapting to local environment to facilitate transmission.^9^ However, these mutations are part of the virus life cycle and should not impact outbreaks.^10^

Zhu et al. extracted viral RNA from respiratory samples of three patients admitted in Wuhan,^2^ and on 7 January 2020, the Chinese Centre for Disease Control and Prevention identified a novel betacoronavirus from one of the samples. They described 85% sequence homology with bat SARS-like CoV which caused the SARS outbreak in 2003.^2,3^ The virus was originally named 2019 novel coronavirus (2019-nCoV) and later changed to SARS-CoV-2. This is the seventh member of the coronavirus family. Of the six previously described CoVs, four cause no more than minimal common cold symptoms in humans. The other two members are SARS-CoV and MERS-CoV.

All CoVs have zoonotic origins, with bats considered the natural reservoir hosts.^3^ In SARS-CoV-2, the sequence identity of pangolin origin CoVs and SARS-CoV-2 is 99%, and this raised the question whether the virus is of pangolin origin.^11^ The natural hosts, namely bats, have a limited inflammatory response as seen by lower interleukin (IL)-1β concentrations and adequate type I interferons, giving rise to a generalized anti-inflammatory milieu and an ability to limit excessive inflammatory response.^12^ Interestingly, this may also explain bats’ relatively long lifespan of 30–40 years.^12^ Type I interferon is known to assist with antiviral control. CoV cannot infect humans unless the virus undergoes mutation and recombination in animal hosts.

SARS-CoV-2 is a highly virulent virus. The original infection distribution in mainland China was found to be 71.45% in patients 30–65 years of age and only 0.35% in patients less than 10 years old.^13^ It is easily transmissible, with initial studies finding that each infected person is capable of infecting another 2.2 individuals.^4^ The virus is stable on plastic and stainless-steel surfaces for up to 72 h,^14^ further enhancing its ability to spread. The relatively long incubation period worsens the situation, as individuals may be asymptomatic carriers for up to two weeks, thereby releasing the virus in their surroundings in a process called ‘viral shedding’.

The most common symptoms of COVID-19 are respiratory. Transmission is via droplets (directly, and on surfaces) and direct contact. SARS-CoV-2 has also been detected in stool which may point to a possible faeco-oral route of transmission.^5,13^

### Risk and comorbidity

Although COVID-19 may present mildly as a flu-like disease or even be asymptomatic, some patients develop significant cardiovascular and other adverse effects. According to present data, 81% of infected individuals have only mild flu-like symptoms and recover within two weeks, 14% have severe symptoms requiring hospitalization and 5% become critically ill.^15^ These differences in presentation are thought to be due to viral load, age and the presence of comorbidities.^12^ As many carriers may be asymptomatic and an underestimated source of transmission,^13^ effective screening and contact tracing are important to identify high-risk individuals, advise self-isolation where transmission risk is high and trace possible cases to limit community spread. Children may also be possible carriers of asymptomatic infection. Lu et al. described that children under 10 years old may be asymptomatic in 15.8% of cases.^16^

The elderly are considered high risk. This may be due to an aging immune system with concomitant lymphopaenia and decreased CD4-count and T-cell regulation.^12^ Elderly people have a propensity to excessive autoimmune and inflammatory responses which may be exacerbated by COVID-19 infection.^12^ Male patients and those with associated comorbidities are also at increased risk.

### Some of the early cases

Risk factors for complicated infection were identified in early studies. Most of these corroborated the increased risk associated with age, male sex and certain comorbidities. The studies also described several laboratory and clinical markers that can be used to determine prognosis.

The first study reported 41 cases admitted early in January 2020 in Wuhan, China. The median age of 49 years (there were no adolescents or children), 73% were male and 66% had exposure to the Huanan Seafood Wholesale market. Of this cohort, 13 (32%) were admitted to an intensive care unit (ICU). Lymphopaenia, increased D-dimer levels, increased partial thromboplastin time (PTT), increased aspartate aminotransferase (AST), increased high-sensitivity troponin-I (hs-TnI) and increased inflammatory cytokines indicated poor prognosis and outcome.^8^

Another early study described 99 hospitalized cases in Wuhan. The average age was 55 years, most were male and 49% had a history of exposure to the Huanan seafood market. Age, obesity and comorbid illness were associated with increased mortality, and concern was expressed about individuals with compromised immune function such as human immunodeficiency virus (HIV), increased age, diabetics or those on immunosuppressive therapy.^17^

A subsequent study of six family members in Shenzhen China who had returned from a visit to Wuhan described the first person-to-person transmission of the virus.^18^

Yang et al. described the clinical course and outcomes in 710 patients with COVID-19 pneumonia including 52 who were critically ill. In the critically ill, the mean age was 59.7 years, 67% were male, 40% had comorbidities and 61.5% had died at 28 days (median time seven days). Non-survival risk was associated with advance age and comorbidities.^19^

Li et al. collected demographic information, exposure history and illness timelines on 425 of the initial confirmed cases in Wuhan. The median age was 59 years and 56% were male. As 55% of cases were linked to the original Huanan Seafood Wholesale Market, this was evident of human-to-human transmission. They described a doubling of cases every 7.4 days and suggested a 14-day observation or quarantine period for exposed persons. They described that each infected person spread the virus to an average of 2.2 people they come in contact with. As none of their cases were children, they postulated that children may be less likely to be infected or have milder symptoms.^4^

Ruan et al. examined factors associated with increased mortality in 150 patients from Wuhan. They collected demographic, clinical and laboratory data and found that age, the presence of comorbidities or secondary infections, and increased cardiac Tn, C-reactive protein (CRP) and IL-6 concentrations were predictors of fatal outcome.^20^

The cytokine storm occurs in a subgroup of patients with severe COVID-19. This is defined as a hyperinflammatory state characterized by a fulminant and fatal increase in inflammatory cytokines with subsequent multiorgan failure. Ferritin concentrations are raised, cytopaenia occurs and approximately half of the patients develop significant pulmonary involvement such as acute respiratory distress syndrome (ARDS).^21^ These findings suggest that in the study by Ruan et al., the mortality in the severely ill patients was probably caused by a cytokine storm.

A study of 195 COVID-19 patients from two hospitals in China, of which one was in Wuhan, found that 54 (27.6%) died in hospital. Increased risk of in-hospital death was associated with older age, organ failure and raised D-dimer levels, and the authors postulated that these could possibly be used on admission to identify patients with a worse prognosis.^3^

Wang et al. examined the epidemiological, demographic, clinical, laboratory, radiological and treatment data on 138 patients hospitalized with COVID-19. They found that 26% of patients needed ICU admission and 4.3% died. The median patient age was 56 years and 54.3% were male. Older age, comorbidities and abnormal laboratory tests including lymphopaenia, increased D-dimer, increased lactate dehydrogenase (LDH) and prolonged PTT were associated with a worse prognosis.^22^

Liu et al. performed a small study on only 12 patients in Shenzhen China, of which eight were males and seven were older than 60 years. They found that laboratory parameters associated with worse prognosis were decreased albumin concentrations, lymphopaenia and elevated CRP and LDH concentrations. They measured angiotensin II concentrations by enzyme-linked immunosorbent assay (ELISA) and found them to be increased. This increased angiotensin II was associated with a high viral load and lung injury suggesting an imbalanced renin angiotensin system (RAS). The authors therefore questioned the possible utility of angiotensin converting enzyme (ACE) inhibitor or angiotensin receptor blocker (ARB) as possible treatment modalities.^23^

The first case diagnosed in USA on 20 January 2020 was a young male patient who had travelled to Wuhan, China. He never visited the Wuhan market or any healthcare facility, and also had no contact with ill people while in the city. The USA’s patient zero initially presented with mild symptoms, but progressed to pneumonia on day nine. His laboratory results showed leucopaenia, mild thrombocytopaenia, elevated creatinine and liver enzymes (alkaline phosphatase [ALP], alanine transferase [ALT], aspartate transferase [AST]).^5^

Arentz studied 21 critically ill COVID-19-infected patients in Washington State with a mean age of 70 years, of which 52% were male. Seventeen (81%) were admitted to ICU, 33% developed cardiomyopathy and the mortality rate was 67%. Again, most were elderly patients, and had comorbidities.^24^

### Renin-angiotensin system and angiotensin-converting enzyme 2

#### RAS normal physiology and receptors

The RAS is an important regulator of blood pressure homeostasis. Renin is a protease secreted by the kidney, which cleaves angiotensinogen, a serpin family protein produced by the liver, to inactive angiotensin I. This is then cleaved to the active angiotensin II by ACE which is secreted by the kidneys and lungs and expressed ubiquitously in the vasculature. Angiotensin II binds angiotensin type 1 (AT1) receptors and causes vasoconstriction and the secretion of aldosterone and antidiuretic hormone with subsequent renal sodium and water reabsorption and raised blood pressure.^25^

When angiotensin II binds AT1 receptor, it leads to an increase in blood pressure as well as inflammation, fibrosis, oxidative stress and vasoconstriction.^26^ This is important for the regulation of cardiovascular, haemodynamic, neurological and endothelial functions.^27^

**Figure 2.**
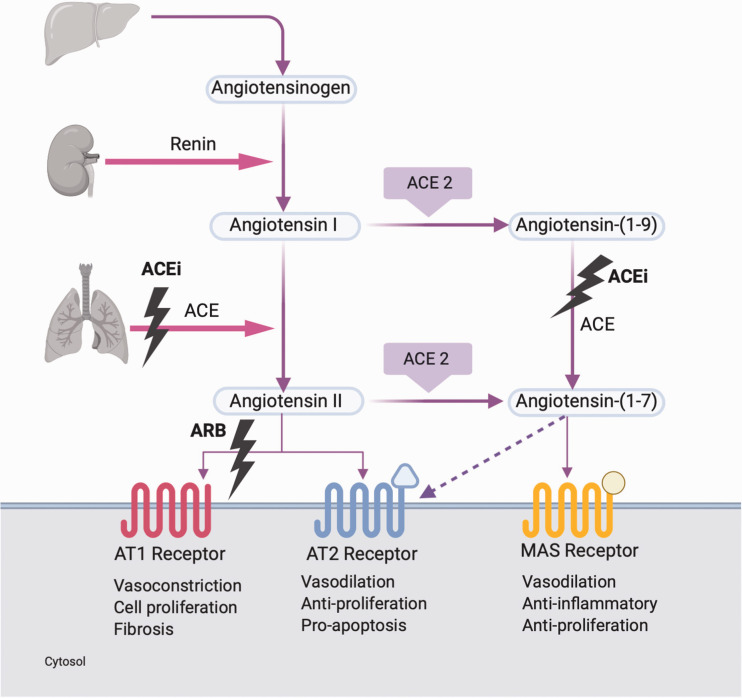
RAS pathway showing various receptors and their effects. Also shown are mechanisms of action for ACE inhibitors and ARB.^27,29^RAS: Renin Angiotensin System; ACEi: Angiotensin Converting Enzyme inhibitor; ARB: Angiotensin Receptor Blocker; AT1: Angiotensin 1; AT2: Angiotensin 2.

*Receptors of the RAS*^27,28^ (Figure 2):

- Angiotensin type 1 (AT1) was originally thought to be the only receptor of angiotensin II and its actions are facilitated via this receptor.^29^ Genetic polymorphisms are associated with hypertension and myocardial infarction. Abnormal activation of AT1 is associated with cardiovascular disease, inflammation and atherosclerosis, endothelial dysfunction, oxidative stress, insulin resistance, cancer and malignant hypertension. AT1 receptor blockers (ARB) are selective antagonists used to treat hypertension and cardiovascular disorders.
- Angiotensin type 2 (AT2) has a 34% homology with AT1 but low expression in adults. Concentrations decrease soon after birth and it is found in low concentrations in the adult cardiovascular system, adrenal glands, kidney, brain, uterine myometrium and skin. Upregulation may occur in physiological stressful conditions such as vascular injury, myocardial infarction and cardiac failure.^29^ Studies in knockout mice demonstrated the development of hypertension. Mutation of the receptor is also associated with mental retardation, and it plays a role in antidiuretic and antinatriuretic functions. As it is also found in brain tissue,^28^ it was investigated as a possible therapeutic target for neuropathic pain. However, the treatment had poor efficacy and many side effects.
- MAS is the receptor for angiotensin (1–7) which is produced by ACE2. Activation of MAS leads to the synthesis of arachidonic acid and activation of nitric oxide (NO) synthase. High expression of MAS is found in the brain, where it is important for cardiovascular regulation. It was also found to play an important role in testicular function where immunolocalization of angiotensin (1–7), and MAS receptors showed impaired spermatogenesis in male infertility.^30^ Knockout mice present with altered heart rate and decreased blood pressure possibly due to NO imbalance and the formation of reactive oxygen species. The protective role of MAS-angiotensin (1–7) has made it a possible drug target.
- Angiotensin type 4 (AT4) receptor is beyond the scope of this review.

## ACE2

A homologue of ACE, namely ACE2 was identified in 2000.^31^ ACE2 and ACE catalytic domains are 42% identical alluding to two genes from a common ancestor.^31^ ACE2 is a type I transmembrane metallopeptidase with its enzymatic domain on the external surface of cells.^25,31–33^ It is involved in regulation of cardiac function and blood pressure control, possibly as a counterpart of ACE.^34^ ACE2 regulates the RAS by modulating endogenous concentrations of angiotensin I and angiotensin II.^25^ It cleaves a single residue of angiotensin I to generate angiotensin (1–9) and a single residue of angiotensin II to generate angiotensin (1–7) (Figure 2). It possibly cleaves other substrates such as apelin which has cardioprotective properties, des-arginine bradykinin which promotes inflammation and neurotensin involved in the regulation of luteinizing hormone and prolactin.^26,31^ Angiotensin (1–9) can also be converted to angiotensin (1–7) by ACE. After binding the MAS receptor, angiotensin (1–7) shifts the balance from vasoconstriction with angiotensin II to vasodilation and gives rise to anti-inflammatory and anti-fibrotic effects, increased NO synthesis and vasodilation.^26^ Inactivation of ACE2 leads to increased angiotensin II concentrations and impaired cardiac contractility.^25^

Although ACE2 mRNA is found in almost all organs, its protein expression is mainly in the lung (airways and type II alveolar cells), heart, kidney and intestine. It is also found in oral and nasal mucosa, skin, lymph nodes, thymus, bone marrow, spleen, liver, testis and brain.^13^ ACE2 has an extracellular domain which is the receptor site for the spike (S) protein of SARS-CoV-2 to gain entry to the cell. It is specifically in lung alveolar epithelial cells and enterocytes that it plays an important role in the entry of CoVs.^34^ ACE2 in enterocytes of the duodenum, jejenum and ileum may explain the virus being found in stool samples and support a possible faeco-oral transmission route.^5,34^ The ACE2 receptor in the tongue and oral mucosa may implicate a possible route of transmission in dental practice.^35^

In keeping with the observation that male individuals are at higher risk for SARS-CoV-2 infection, it is important to note that the ACE2 gene has been mapped to the X-chromosome.^25^ Females have two copies of the ACE2 gene, and males only have one copy which is a possible theory why males are at higher risk.

There are two forms of ACE2. The full-length ACE2 is found in cell membranes and consists of a transmembrane anchor and an extracellular domain which is the receptor site for the spike (S) protein of SARS-CoV-2. The second form is a soluble form which is shed into the circulation. This form of ACE lacks membrane anchors and circulates in low concentrations.^36^ Circulating levels do not reflect issue levels. It has been proposed that the soluble form of ACE2 may inhibit the binding of SARS-CoV-2 to membrane-bound ACE2 and therefore proposed a soluble recombinant of ACE2 as a potential treatment for COVID-19.^36^

### ACE2 in inflammation and disease

When angiotensin II binds to the AT1 receptor, vasoconstriction, inflammation, fibrosis and proliferation follow leading to hypertension, cardiac fibrosis, thrombosis and ARDS. Binding of angiotensin (1–7) to MAS receptor leads to vasodilatation, anti-apoptotic, anti-fibrotic and anti-proliferation activity and a reduction in blood pressure, decreased cardiac fibrosis, decreased thrombosis and decreased ARDS.^37^

When AT2 is bound, NO is released, which is an anti-inflammatory vasodilator, reduces platelet aggregation and may facilitate the insulin action.^29^ Therefore, angiotensin II binding of AT1 is an inflammatory mediator via reactive oxygen species (ROS) and nuclear factor kappa β (NFKβ) and provides a mitogenic stimulus for smooth muscles leading to oxidative stress. This is a common feature of atherosclerosis, diabetes mellitus, hypertension and hyperlipidaemia. Stimulation of AT1 also leads to upregulation of adhesion molecules and interferes with insulin signalling leading to insulin resistance. ACE inhibitors and ARB therapy were found to decrease circulating CRP, and this is thought to be due to decreased angiotensin II stimulation of AT1.

Disruption of ACE2 in mice leads to severe cardiac contractility defects, increased angiotensin II concentrations and the expression of hypoxia-induced cardiac genes. When ACE was also decreased, this cardiac phenotype was rescued. However, loss of ACE2 did not affect blood pressure or kidney development in these mice, and it was postulated that ACE could potentially compensate for loss of ACE2. Yet, even when these mice were treated with ACE inhibitor, there was a similar reduction in blood pressure as in wild type; therefore, ACE2 has no apparent direct effect on blood pressure.^25^

A strong association between RAS and cardiovascular disease, neurodegenerative disorders and acute lung injury was demonstrated in mice with acid aspiration lung injury. In severe acute lung failure, a significant downregulation of ACE2 was noted. The expression of ACE was unchanged leading to a marked increase in angiotensin II, as its formation is upregulated by ACE. Therefore, ACE promotes disease through increased angiotensin II, and ACE-deficient mice were partly protected against acid-induced lung injury. Recombinant ACE2 and AT2 receptor expression protected mice from severe lung disease in this scenario.^38^ The inactivation of ACE on an ACE2 knockout background rescued the mice from severe lung failure. Therefore, it was postulated that ACE promotes acute lung injury, and ACE2 alleviates it.^38^

Patel and Verma found that high pulmonary ACE2 concentrations protect against lung injury.^39^ The loss of ACE2 expression in acute lung injury led to leaky pulmonary blood vessels through AT1 receptor stimulation. However, hydrostatic oedema could not be excluded as a causative factor, emphasizing the need for further studies on the local effects of angiotensin II in lungs.^38^

Pathology of the RAS is associated with pulmonary hypertension and pulmonary fibrosis.^40^ Angiotensin II upregulates the expression of profibrotic cytokines leading to pulmonary fibrosis and severe inflammation with increased vascular permeability. Treatment with ACE inhibitors and ARB may attenuate this.^40^ Animal models of ARDS found reduced ACE2 concentrations, and treatment with exogenous ACE2 alleviated the symptoms.^37^

Further studies found that the loss of ACE2 rendered subjects susceptible to heart failure and postulated that increasing ACE2 concentrations were cardioprotective. Loss of ACE2 also led to age-dependant cardiomyopathy, pulmonary, cardiac and renal injuries.^33^

Diabetes mellitus, a major comorbid risk factor in COVID-19, is associated with increased ACE2 expression. This may explain why patients suffering from diabetes have greater susceptibility to SARS infection.^33^

ACE2 is involved in the possible protective arm of the RAS. The ACE2/angiotensin (1–7)/MAS axis counterbalances the ACE/angiotensin II/AT1 axis which is associated with vasoconstriction, inflammation, cell proliferation, hypertrophy, fibrosis and tissue modelling. Therefore, ACE2 protects against RAS-induced injuries by decreasing angiotensin II and generating angiotensin (1–7), which increases substrate availability of the protective axis.^41^ ACE2 knockout mice and those on ACE2 inhibitors had increased susceptibility to myocardial infarction, hypertension, angiotensin II-induced myocardial hypertrophy, microvascular complication, inflammation, fibrosis, diastolic and systolic dysfunction and oxidative stress.

### Medication that influences ACE2 concentrations

ACE inhibitors block ACE and ARB block the action of angiotensin II on ATI receptors. Both lead to an increased ACE2 activity.^42^ The drugs may alleviate inflammation, decrease endothelial dysfunction and increase insulin sensitivity.^29^ The ARBs have more potential to block the RAS than ACEI, as about 40% of angiotensin II is formed via non-ACE pathways in humans.^29^ ARBs are known to have less side-effects than ACE inhibitors.

Another area of interest is peroxisome proliferator-activated receptor-gamma (PPAR-γ). Insulin resistance is strongly associated with hypertension. ACE2 expression is increased with the use of PPAR-γ agonists.^26^ PPAR-γ agonists, thiazolidinediones, attenuate the development of hypertension and improve endothelial dysfunction in angiotensin II-infused rats.^43^ These rats had decreased AT1 expression; therefore, the effects were most likely mediated through AT2 receptor activation.^44^ However, PPAR-γ agonists were shown to increase the risk of congestive heart failure and possibly myocardial infarction.

Other medications that have an effect on ACE2 concentrations include mineralocorticoid antagonists such as the aldosterone receptor antagonist spironolactone, which possibly increases ACE2 concentrations in macrophages. This was not the case in the heart tissue of patients suffering from chronic heart failure.^45^ Statins possibly augment ACE expression in heart and kidney tissue, and may also bring about epigenetic changes in ACE2 gene, but more work is needed to determine the exact mechanism of these findings.^26^

Presently, there is still a paucity of evidence on how ACE inhibitors or ARB may impact the ACE2/angiotensin (1–7) pathway in the lung, heart and brain, and ongoing research in this field is needed.

### ACE2 and COVID-19

Human ACE2 is a functional receptor essential for cellular entry of CoVs and is the same receptor used by SARS-CoV to gain access to cells.^32^ Kuba et al. provided the first genetic proof that ACE2 was a crucial receptor for SARS-CoV *in vivo*.^46^ When ACE2 knockout and wildtype mice were infected with SARS-CoV, the knockout mice had very low levels of viral replication in their lungs. In the wildtype mice, SARS-CoV infection was associated with lung failure which was attenuated by treatment blocking the RAS. They postulated that SARS-CoV infection downregulated ACE2 concentrations, possibly explaining the decline in lung function observed with viral infection.^46^

**Figure 3.**
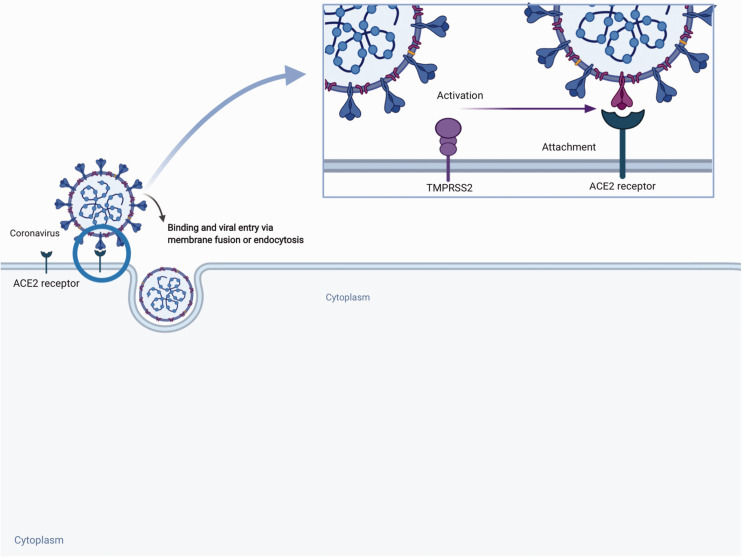
Entry of SARS-CoV-2 into host cells using ACE2 Receptor and TMPRSS2 to gain entry.^51^SARS-Co-V-2: Severe Acute Respiratory Syndrome-Coronavirus-2; ACE2: Angiotensin Converting Enzyme 2; TMPRSS2: transmembrane protease, serine 2.

The spike (S) proteins of CoVs facilitate viral entry into the target cells by attaching to ACE2 as an entry receptor.^32,47^ SARS-CoV-2, like SARS-CoV, has a viral envelope studded with spikes consisting of glycoproteins made up by two subunits, namely S1 and S2. Subunit S1 binds the cell surface bound ACE2 and subunit S2 fuses with the cell membrane.^42^ The cellular serine protease, TMPRSS2, is essential for S-protein priming and facilitates viral cell entry.^47^ TMPRSS is expressed on SARS-CoV target cells.^48^ After subunit S1 of the spike binds to membrane bound ACE2, TMPRSS2 activates the spike and cleaves the ACE2 receptor. TMPRSS2 also acts on the S2 subunit, causing an irreversible conformational change facilitating fusion of the viral membrane with the host cell membrane and endocytosis.^13,47^ Therefore, both ACE2 and TMPRSS2 are essential for cellular entry. SARS-CoV-2 binds human ACE2 with 10–20-fold higher affinity than SARS-CoV.^13^ This may be due to a more compact virus structure enabling better receptor affinity and enhanced ability to be internalized. Figure 3 shows the cellular entry of the SARS-CoV2.

The binding of SARS-CoV-2 may decrease ACE2 concentrations and increase the angiotensin II to angiotensin (1–7) ratio which may exacerbate pulmonary injury and tissue fibrosis.^26^ Angiotensin (1–7) may provide resilience from the development of pulmonary failure in COVID-19 further supporting this finding.^49^

Oudit et al.^33^ determined that SARS-CoV infection has a detrimental effect on cardiac function and led to cardiac dysfunction, arrhythmias and death. They measured ACE2 concentrations in mice infected with human strain SARS-CoV and in hearts obtained from autopsy of patients who died from SARS infection. They found that SARS-CoV infection led to a decrease in ACE2 expression in the myocardium of mice with subsequent myocardial dysfunction. SARS-CoV mRNA expression was found in 35% of the autopsy heart samples, which was associated with decreased ACE2 protein expression. Therefore, they postulated that SARS-CoV leads to cardiac dysfunction by downregulating myocardial ACE2, possibly due to a cytokine-mediated downregulation of ACE2 mRNA.^33^

The gastrointestinal system is also not spared from the effects of SARS-CoV-2 infection. Xiao et al. found that ACE2 was abundant in the glandular cells of gastric, duodenal and rectal epithelia.^50^ Hamming et al. described ACE2 in the small intestine, further underlining the importance of SARS-CoV-2 involvement in the gastrointestinal system.^34^

An interesting observation is the difference in infection rate and adverse outcomes in adults and children. Although there have been reports of less COVID-19 infection in children, there is a lack of evidence that ACE2 changes with age.^37^ A possible hypothesis is that children are less susceptible to COVID-19 infection due to cross-protective antibodies from multiple upper respiratory tract infections and that their lower respiratory tracts have less ACE2.^49^ Limited studies and data in children are available, and conclusive evidence to support postulations that children are less likely to be adversely affected by SARS-CoV-2 infection is lacking.

It is clear from statistical data that older people, especially men are more affected by COVID-19. Increased ACE2 have been described in older individuals and in males,^52^ which could possibly be linked to the worse outcome in this population group.

### ACE inhibitors and ARB in COVID-19

As yet, there is no evidence of causal relationship between ACE2 activity and SARS-CoV-2 mortality. Also, ACE2 concentrations do not correlate with the degree of disease severity.^53^ Activation of the RAS is associated with endothelial dysfunction and multiple organ injuries; therefore, theoretically, treatment focusing on manipulation and control of this system may be beneficial.^37^

COVID-19-infected patients have a worse prognosis when they suffer from comorbidities, especially cardiovascular. RAS inhibitors have been shown to have a protective effect on the kidney and myocardium, and it may therefore be dangerous to withdraw treatment in these patients. A higher risk of relapse in patients with cardiovascular or renal disease has also been demonstrated.^54^

Angiotensin II concentrations were found to be increased in COVID-19 infection and positively correlated with viral load and lung injury.^23^ Increased ACE2 concentrations may possibly lead to a greater susceptibility for COVID-19 infection but may also be cardioprotective. This led to a crossroad in clinical decision-making: RAS blocking treatment may increase ACE2 and facilitate virus entry, but the beneficial effects it has in patients suffering from comorbid conditions cannot be ignored.

A lot can be learned from studies involving other respiratory viruses where the use of ACE2-lowering treatment was investigated. A study on patients with novel influenza A (H7N9), a virus of avian origin from eastern China in 2013, found that more than 70% developed ARDS and had a high mortality. Patients with markedly elevated angiotensin II concentrations had poorer disease outcome with a predictive value higher than CRP. The authors speculated that recombinant ACE2 and angiotensin II-lowering treatment such as ACE inhibitors and ARB could be beneficial.^51^

Avian influenza A (H5N1) primarily infects poultry and birds, but since 2003 has also been infecting humans. Increased angiotensin II and downregulation of ACE2 was linked to infection severity and mortality. When mice infected with H5N1 were given recombinant ACE2, the virus-induced lung injury was ameliorated.^55^

The use of intravenous ACE inhibitors and ARB in experimental animals increases ACE2 expression in the cardiopulmonary circulation systems, causing a possible increased risk for SARS-CoV-2 infection.^56^ Severe COVID-19 outcomes were described in patients with existing hypertension, coronary artery disease, diabetes and chronic kidney disease.^57^ The question was asked whether their current treatment regimens were increasing their risk for infection.

Theoretically, if ACE2 activity in cell membranes decreased, it should reduce the ability of SARS-CoV-2 to enter cells. But, this also decreases the protective effects of ACE2 in various organ systems where ACE2 is expressed. Angiotensin II may contribute to organ damage in COVID-19,^58^ and concentrations were found to correlate with viral load and lung injury.^23^ Downregulation of ACE2 in the lung tissue may facilitate neutrophil infiltration, leading to angiotensin II accumulation and RAS activation.^54^

When COVID-19 binds ACE2 and enters the cell, the expression of ACE2 is downregulated. This downregulation essentially ‘removes the brakes’ from angiotensin II and possibly induces local activation of immune cells. In lungs, decreased ACE2 in alveolar type II cells leads to surfactant deficit and the development of lung injury.^59^ Although an increase of ACE2 may lead to higher cell infection rates and proliferation of the virus, it may be beneficial in patients with lung injury and withdrawal may be harmful especially in high-risk patients.^54^

In a study on acid-induced lung injury in mice, ACE2 downregulation by SARS-CoV worsened lung injury. The induced lung injury improved with the use of ARBs, and the findings are suggestive that SARS-CoV exacerbates lung injury by decreasing ACE2, and that it is also reversible by ARBs.^46^

An increase in ACE2 in heart tissue may be cardioprotective. Therefore, SARS-CoV-2 could possibly influence the fine balance between angiotensin II and angiotensin (1–7), whereas treatment could block RAS and protect the heart and other organs.^37^

Cardiac tissue function may be compromised by the increase in angiotensin II to angiotensin (1–7) ratio. Loss of ACE2 in the central nervous system, specifically the brain stem cardiovascular centre, may cause an increased sympathetic drive, changes in baroreflex and worsening of hypertension.^26^ Loss of ACE2 in the vasculature can contribute to endothelial dysfunction and inflammation and exacerbate existing atherosclerosis and diabetes.

There have been concerns that increased ACE2 expression in heart, brain and urine after treatment with ARB may increase an individual’s risk of infection, but very little evidence is available showing significant changes in serum of pulmonary ACE2 concentrations. However, the significance of ACE2 expression and its effect on COVID-19 pathogenesis and mortality are uncertain.^39^

Therefore, do the benefits outweigh the risks? A joint statement issued on 17 March 2020 found that no experimental or clinical evidence demonstrating beneficial or adverse outcomes in COVID-19 patients using ACE inhibitors or ARB was available and that urgent additional research is needed. It stated that treatment should not be discontinued if patients become infected with SARS-CoV-2.^60^

### Potential COVID-19 treatment involving ACE2 system

Since the early stages of the COVID-19 pandemic, emphasis has been on finding possible treatment modalities. A number of possible treatment targets were identified with most based on previous studies conducted on SARS-CoV, specifically involving the ACE2/angiotensin (1–7) pathway.

#### Decreasing ACE2

Downregulation of ACE2 was proposed as a possible treatment to reduce susceptibility to COVID-19 infection. However, the ensuing relatively unopposed action of angiotensin II could lead to worsening hypertension, inflammation, thrombosis and adverse lung and cardiac outcome.^41^ A study by Kuba et al. determined that infection with SARS-CoV downregulates the expression of ACE2. These low ACE2 concentrations were associated with lung oedema and acute lung injury, which contributed to the severe lung pathology in patients already on ACE inhibitors at the time of SARS-CoV infection. They concluded that modulating RAS may therefore be useful in treating infection.^46^

Some studies also questioned the use of anti-ACE2 antibodies to inhibit viral replication of SARS-CoV. These could possibly inhibit virus-induced cytopathicity in a dose-dependent manner.^32^

#### Increasing ACE2

Trials are underway to evaluate the safety and efficacy of recombinant human ACE2 and the ARB losartan in COVID-19-infected patients. Infusion of recombinant human ACE2 may act as a decoy to interfere with viral replication. Soluble ACE2 could potentially bind SARS-CoV and thereby prevent the binding of the viral spike protein to full length membrane-bound ACE2^36^ reducing viral infection of cells.^36^ Recombinant ACE2 was shown to reverse lung injury in SARS-CoV-infected patients.^55^ Wang et al. found that ACE inhibitors and ARB offer partial cardiovascular protection by increasing ACE2, and that IV recombinant ACE2 may be beneficial in preventing pulmonary arterial hypertension and acute lung injury.^41^

However, circulating ACE2 concentrations are usually very low to undetectable and do not accurately represent the amount of membrane-bound ACE2 and would not be sufficient to sequester SARS-CoV-2 in the circulation.^26^ A proposed clinical trial with recombinant ACE2 was withdrawn due to the unknown effects and the possibility of the drug inducing significant hypotension.^26^

ACE2 interaction with SARS-CoV is important in the development of SARS-associated cardiomyopathy, as viral RNA was found in the myocardium on postmortem examination. The associated cardiac injury due to reduced expression of ACE2 possibly played a major role in the morbidity and mortality of patients infected with SARS-CoV. Recombinant ACE2 was found to normalize angiotensin II concentrations, suggesting that it may be beneficial to prevent organ damage caused by SARS-CoV. The question thus arose if it is possible and beneficial to balance RAS control to reduce infection and prevent organ damage in COVID-19-infected patients.^33^

#### Targeting TMPRSS2

Protease inhibitors against TMPRSS2 block viral entry *in vitro* by inhibiting the entry of SARS-CoV-2 into cells.^47^ Camostat mesylate, a serine protease inhibitor used in Japan to treat chronic pancreatitis, inhibits TMPRSS2 and can block entry of SARS-CoV-2 into bronchial epithelial cells *in vitro*.^47,48^

#### Angiotensin II

Many patients with severe COVID-19 infection admitted to ICU are in septic shock. As most angiotensin I to angiotensin II conversion by ACE occurs in the lungs, significant lung injury may decrease this. Decreased ACE function was found to be a predictor of mortality. Angiotensin II, a novel vasopressor agent, may be beneficial in this patient population.

Strategies to decrease ACE2 may possibly attenuate SARS-CoV-2 infectivity. This may happen in a number of ways. Firstly, endogenous angiotensin II theoretically prevents infection by binding the ACE2 during its degradation and therefore may compete with SARS-CoV-2 to bind to the receptor. Secondly, binding of angiotensin II to AT1 causes internalization and downregulation of ACE2. And lastly, angiotensin II causes AT1-dependent destruction of ACE2 further decreasing the virus’ ability to enter cells. Angiotensin II is not yet available for use commercially, but due to the number of critically ill patients, and the promising outcome of the drug, it has been made available for compassionate use in Italy, Germany and UK.^61^

#### Angiotensin (1–7)

Another focus area is the use of angiotensin (1–7) as a novel treatment for COVID-19.^26^ Experimental studies found that angiotensin (1–7) reduced the acute inflammatory response and subsequent fibrosis in acid-induced ARDS in mice.^62^ Angiotensin (1–7) protected the lung against inflammation and fibrosis, inhibited alveolar cell apoptosis, attenuated endothelial cell activation, loss of barrier function and oedema, and decreased synthesis of pro-inflammatory and pro-fibrotic cytokines. The authors therefore recommended that the concentrations of angiotensin (1–7) should be increased to protect COVID-19-infected patients.^62^

## Conclusion

The effects of ACE2 on COVID-19 infections need to be further evaluated and randomized control trials are necessary to obtain the highest level of evidence. Although ACE2 has beneficial effects by regulating the protective arm of RAS, some authors postulate that high levels make patients more susceptible to COVID-19 infection. The RAS has numerous attractive therapy targets for COVID19 infection. Trials are needed to evaluate the use of ACE inhibitors and ARB’s in COVID-19 patients.
